# Congenital Cervical Teratoma:Anaesthetic Management (The EXIT Procedure)

**Published:** 2009-12

**Authors:** Ferruh Bilgin, Nedim Cekmen, Yavuz Ugur, Ercan Kurt, Sadettin Güngör, Cuneyt Atabek

**Affiliations:** 1 Gülhane Military Medical Faculty, Department of Anesthesiology and Reanimation; 2 Gülhane Military Medical Faculty, Department of Anesthesiology and Reanimation; 3 Gülhane Military Medical Faculty, Department of Anesthesiology and Reanimation; 4 Gülhane Military Medical Faculty, Department of Anesthesiology and Reanimation; 5 Department of Obstetrics and Gynecology; 6 Department of Pediatric Surgery

**Keywords:** Cervical teratoma, Mass in the neck, Extrauterine intrapartum treatment procedure.

## Abstract

**Summary:**

Ex utero intrapartum treatment (EXIT) is a procedure performed during caesarean section with preservation of fetal-placental circulation, which allows the safe handling of fetal airways with risk of airways obstruction. This report aimed at describing a case of anaesthesia for EXIT in a fetus with cervical teratoma. A 30-year-old woman, 70 kg, 160 cm, gravida 2, para 1, was followed because of polyhydramniosis diagnosed at 24 weeks’ gestation. During a routine ultrasonographic examination at 35 weeks’ gestation, it was noticed that the fetus had a tumoral mass on the anterior neck, the mass had cystic and calcified components and with a size of was 10 × 6 ×5 cm. The patient with physical status ASA I, was submitted to caesarean section under general anaesthesia with mechanically controlled ventilation for exutero intrapartum treatment (EXIT). Anaesthesia was induced in rapid sequence with fentanyl propofol and rocuronium and was maintained with isoflurane in 2.5 at 3 % in O and N O (50%). After hysterotomy, fetus was partially released assuring uterus-placental circulation, followed by fetal laryngoscopy and tracheal intubation. The infant was intubated with an uncuffed, size 2.5 endotracheal tube. Excision of the mass was performed under general anaesthesia. After surgical intervention, on the fourth postoperative day, the infant was extubated and the newborn was discharged to the pediatric neonatal unit and on the seventh day postoperatively to home without complications. Major recommendations for EXIT are maternal-fetal safety, uterine relaxation to maintain uterine volume and uterus-placental circulation, and fetal immobility to help airway handling. We report one case of cervical teratoma managed successfully with EXIT procedure.

## Introduction

Teratomas are germ cell tumour derived from pluripotential cells and consisting elements of different types of tissue from one or more of the three germ cell layers^1^,^2^. Commonly found in the sacrococcygeal region, teratomas may originate from any part of the body. In 3-5 % of cases, the teratoma may arise from the head and neck region, with mostly anterior and lateral placement. The mortality of newborn increases in proportion to the size of lesion without treatment. Early diagnosis with imaging methods and planning of delivery are the most important steps of the treatment at the postpartum period^1^–^4^.

A careful anaesthetic management is necessary to provide oxygenation of fetus during cesarean section and to maintain a patent airway after delivery. This may be planned, in the case of antenatally diagnosed lesions, when the pediatric anaesthetist is part of a multidisciplinary team involved in an Extra Uterine Intrapartum Treatment (EXIT) or when a neonate is undergoing elective excision in the early neonatal period as definitive treatment. Alternatively the anaesthetist may be called upon urgently to secure a compromised airway immediately postpartum when no antenatal diagnosis has been made. Furthermore, after elective surgical excision, airway compromise is possible, which may again require anaesthetic intervention. The EXIT procedure allows the fetoplacental circulation to continue during cesarean section and it is also helpful to apply invasive instrumentation to the neonate. Initially, only the infant's head and shoulders are delivered, thus maintaining uteroplacental blood flow.After the infant's airway is secured, umbilical cord is clamped and delivery of the infant is completed^2^–^5^.

Our case was diagnosed with cervical teratoma at the prenatal period and an EXIT procedure was planned because of expected difficulty of airway instrumentation. This procedure was successfully applied and the infant was intubated during oxygenation.

## Case

**Fig 1:**
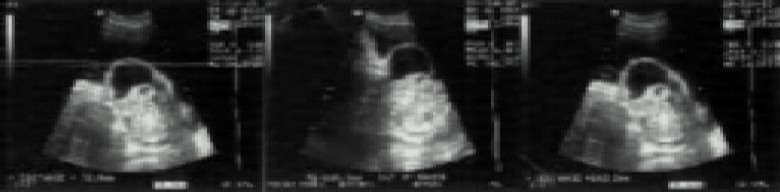
Intrauterine appearance of fetus in ultrasonographic imaging

**Fig 2:**
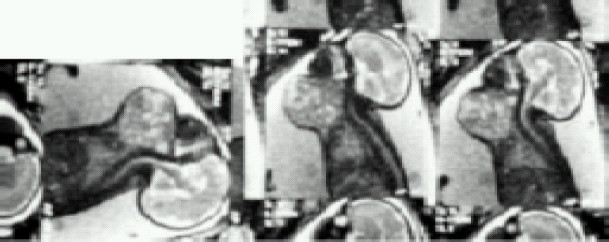
Intrauterine appearance of fetus in CT

A 30-year-old woman, 70 kg, 160 cm, gravid 2, para 1, was followed because of polyhydramniosis diagnosed at 24 weeks’ gestation. During a routine ultrasonographic examination at 35 weeks’ gestation, it was noticed that the fetus had a tumoral mass on the anterior neck, which was protruding outwards under the jaw bone, pushing the floor of mouth upward, oblit- erating the trachea and esophagus and extending to the sternal notch (Fig 1). The mass had cystic and calcified components and with a size of was 10 × 6 ×5 cm. The blood supply of the mass was poor. All of the findings were confirmed with CT scan too (Fig 2). The woman and her husband had no significant medical history.

The case was diagnosed with cervical teratoma and evaluated with a team including anaesthesiologists, gynecologists, neonatal intensive care specialists and pediatric surgeons because of possibility of respiratory distress due to an airway obstruction at postnatal period. It was decided that the infant would be intubated immediately after the delivery which would be a cesarean section under elective conditions and the cervical mass would be excised if necessary. After information and explanation about the anaesthetic procedure, without anaesthetic background, physical status ASA I, was submitted to cesarean section under general anaesthesia with mechanically controlled ventilation for exutero intrapartum treatment (EXIT).

**Fig 3:**
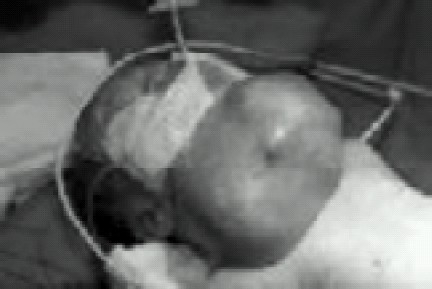
Postnatal apparence of newborn

Patient was placed in the supine position and uterus was displaced to the left with the aid of Crawford's wedge. Monitoring consisted of cardio- scope at DII lead, noninvasive blood pressure, pulse oximetry, capnography and neuromuscular block evaluation by acceleromyography. Patient was premedicated with intravenous metoclopramide (10 mg) and ranitidine (50 mg), 30 minutes before anaesthesia. Upper limb vein was catheterized in the operating room at room temperature with disposable 14G catheter for volume replacement and drug administration. General anaes- thesia was induced in rapid sequence, oxygenation with 100 % oxygen under mask, intravenous fentanyl (250 µg), propofol (140 mg) and rocuronium (50 mg), Sellik maneuver and tracheal intubation. Anaesthesia was maintained with isoflurane in 2.5 % concentration at 3% through gauged vaporizer and administered in mixture of O2 and N2O (50 %). Maternal arterial oxygen saturation remained at 100 % during the entire procedure. Fetal was partially released (head, shoulders and upper limbs) after hysterotomy and, after assuring fetal placental circulation. The intubation procedure was started while uteroplacental blood flow was still con- tinuing. During intubation, Cormack and Lehane direct laryngoscopy score was evaluated as grade 4 (Fig. 3). Oxygen peripheral saturation and fetal pulse frequency were continuously evaluated during the procedure with the aid of pulse oximetry and sterile sensor placed on right hand, which were maintained in approximately 70 % and 108 bpm, respectively. After tracheal intubation SpO2 has increased to 92 % and pulse frequency was maintained in approximately 100 bpm. Fetus was then totally released, umbilical cord was clamped and uterus was continuously sutured. Isoflurane concentration was gradually decreased and oxytocin (20 UI) continuous infusion and intravenous methyl-ergonovine (0.2 mg) were administered to reestablish uterine tone. Maternal end-tidal carbon dioxide ranged from 25 to 35 mmHg and maternal systolic blood pressure was over 100 mmHg all through the surgery. The infant was intubated with an uncuffed, size 2.5 endotracheal tube. Fetal airway handling and tracheal intubation were performed in 3 minutes, the cord was clamped after the correct endotracheal tube positioning was confirmed and the airway was secured, with total surgery duration of 80 minutes. The infant was transferred to another operation room where the resection of the cervical mass would be performed by pediatric surgeons. Anaesthesia was maintained with routine procedure. At the operation, the cervical mass was observed to be attached in places to anterior group muscles of the neck and pushed the larynx posterolaterally. Newborn presented Apgar scores of 6 and 9 at 1 and 5 minutes, respectively, being referred to neonatal ICU with spontaneous ventilation. Parturient was referred to the post-anaesthetic recovery unit conscious and hemodynamically stable. It was concluded that the mass originated from the left lobe of thyroid gland. The mass was removed with careful disection from larynx and trachea and resected completely. The pathologic examination revealed an immature teratoma, grade I.

After surgical intervention, on the fourth postoperative day, the infant was extubated and the newborn was discharged to the pediatric neonatal unit and on the seventh day postoperatively to home without complications.

## Discussion

Congenital cervical teratomas are uncommon lesions with on incidence of 3 % in all teratomas of childhood, but they have poor prognosis^1^. Cervical teratomas are rare congenital tumors derived from all three germ cell layers. The vast majority are histologically benign, but the significant size they may attain can potentiate life-threatening upper airway obstruction. Most of the lesions are benign, but they may cause mortality due to an airway obstruction by a mass effect. Airway obstruction at birth is life threatening and associated with a high mortality. In giant fetal neck masses, this mortality is usually associated with a delay in obtaining an airway and the inability to effectively ventilate the infant. This delay can result in hypoxia and acidosis and, if the delay is greater than five minutes, anoxic brain injury may occur. Early diagnosis and planning are essential. Without a team, there is little hope for fetal survival; Mortality rates change in range of 80–100 % 2. The diagnosis may be achieved antenatally with ultrasonograpic examination. Polyhydramniosis accompanies in 20-30 % of cases. The rate of stillbirth is reported in 17 % of cases^3^. The mortality rate is reported to be 9-17 % in the surgically cases treated^4^.

Jordan and Gauderer^5^ rewieved 163 congenital cervical teratoma cases and classified them in three groups based on the survey. They are; a- Premature infants b- Newborn with respiratory distress c- Newborn without respiratory distress. The mortality rates of the groups were reported as 100 %, 43.4 % and 2.7 %. Our case was in the newborn with respiratory distress group defined as group b.

Early diagnosis and multidisciplinary management are extremely important. Formation of a multispecialty team and use of the EXIT procedure is essential for survival of the neonate. The EXIT procedure was described first in 1990 by Zerella and Finberg for tracheal obstruction in a neonate^6^ and has been refined by Mychaliska et al.^7^ In the EXIT procedure, deep inhalational anaesthesia ensures uterine relaxation, which is crucial to preserving uteroplacental gas exchange. The EXIT procedure allows for the management of newborns diagnosed antenatally with extrinsic (lymphan- giomas, teratomas) or intrinsic (laryngeal atresia, congenital upper airway obstruction syndrome) obstructive malformations. EXIT, for maintaining uterus-placental circulation and, as a consequence, adequate fetal oxygenation during the time needed for airway handling (laryngoscopy, bronchoscopy, tracheal intubation or tracheotomy), is a common procedure in such situ- ations^8^,^9^.

The most important step of the management is providing a safe airway for newborn while the uteroplacental flow is continuing during delivery. Shih et al^8^ reported that they secured the airway via tra-cheostomy for a case with pretracheal teratoma at the 51 minute of the delivery Mychaliska et al^7^ proposed that this period may be prolonged to 60 minutes with deep inhalation anesthesia. In our case, we have intu- bated the newborn at the fifth minute after skin incision. In the literature there are different methods for the main- tenance of uteroplacental blood flow. Prophylactic indomethaeine may be applied to the pregnant for tocolysis. Nitroglycerin and terbutaline may be used to reduce the uterine tonus perioperatively. High dose in- halation anesthetic agents (2-3 MAC) may be applied to achieve uterine relaxation^9^. In our case, we achieved uterine relaxation via inhalation of 2-3 % sevoflourane deep inhalation anaesthesia and so we gained extra time for securing the airway while the uteroplacental blood flow continued.

Although the uterine tonus is reduced, it is important to maintain sufficient maternal systolic blood pressure for the continuation of placental blood flow. For this purpose, efficient treatment with relaxant agents, vasopressors and fluid rescucitation is necessary^10^. Angiotensin II infusions are useful to maintain the systolic blood pressure over 100 mmHg or the blood pressure may be increased with small boluses of iv ephedrine when blood pressure declines. In our case, the heat loss was minimized, the umbilical cord was not endangered and premature placental separation was prevented with delivery of only head and shoulders.

It is emphasized that placement (occupation) of the fetus at the same level with the placenta may pre- vent excessive hydrostatic pressure over the fetoplacental circulation and also prevent premature placental separation^11^. We have paid attention to this point in our case. The surgical excision of the mass is the most appropriate treatment for congenital cervical teratomas. In our case, the mass was completely re- moved from surrounding tissues. Respiratory distress persisted until the fourth day postoperatively, and the newborn was extubated on the fourth day.

Congenital cervical teratomas are one of the rare congenital abnormalities which necessitate early ante- natal diagnosis and multidisciplinary approach. Other- wise, the mortality rate will be high. We have already made our crucial preparations for the case who was diagnosed at the antenatal period and planned a cesarean section. In this manner, we have secured the newborn's airway with suitable interventions. The EXIT procedure allows therapeutic interventions on the neonate while maintaining fetoplacental circulation and thereby maintaining oxygenation. These cases highlight the possible airway scenarios that may confront the anaesthetist in the immediate postpartum, elective surgery and postoperative stages and the variety of techniques that may be employed in order to overcome the potential difficulties encountered.

In conclusion, major recommendations for EXIT are maternal-fetal safety, uterine relaxation to maintain uterine volume and uterus-placental circulation, and fetal immobility to help airway handling. Formation of a multispecialty team and use of the EXIT procedure is essential for survival of the neonate. We report one case of cervical teratoma managed successfully with EXIT procedure.
